# Safety of tacrolimus during lactation: an exploratory retrospective analysis in a small Japanese cohort

**DOI:** 10.1186/s13006-026-00820-1

**Published:** 2026-02-07

**Authors:** Kazushi Yashima, Taku Obara, Fumiko Matsuzaki, Chihiro Suzuki, Mina Koyama, Moeko Hosono, Aoi Noda, Shinichi Sato, Tetsuro Hoshiai, Takushi Hanita, Masatoshi Saito, Nariyasu Mano

**Affiliations:** 1 Department of Pharmacy, Tohoku Medical and Pharmaceutical University Wakabayashi Hospital, 2-29-1 Yamato-machi, Wakabayashi-ku, Sendai, Japan; 2 Department of Pharmaceutical Sciences, Tohoku University Hospital, 1-1 Seiryo- cho, Aoba-ku, Sendai, 980-8574 Japan; 3 Department of Molecular Epidemiology, Tohoku University Graduate School of Medicine, 2-1 Seiryo-cho, Aoba-ku, Sendai, Japan; 4 Division of Preventive Medicine and Epidemiology, Tohoku Medical Megabank Organization, Tohoku University, 1-1 Seiryo-cho, Aoba-ku, Sendai, Japan; 5 Department of Pediatrics, Tohoku University Graduate School of Medicine, 1-1 Seiryo-cho, Aoba-ku, Sendai, Miyagi Japan; 6 Department of Obstetrics and Gynecology, Tohoku University Graduate School of Medicine, 1-1 Seiryo-cho, Aoba-ku, Sendai, Miyagi Japan

**Keywords:** Autoimmune diseases, Breastfeeding, Japan, Tacrolimus

## Abstract

**Background:**

Tacrolimus is a calcineurin inhibitor immunosuppressant widely used in organ transplantation and the management of autoimmune diseases. Tacrolimus is generally considered to be safe for women who are breastfeeding; however, pharmacokinetic differences related to genetic background have been reported, and clinical data focusing on Japanese women during lactation remain scarce. As such, this study evaluated the safety of tacrolimus in Japanese women diagnosed with autoimmune diseases during breastfeeding.

**Methods:**

This exploratory retrospective analysis was conducted at a single tertiary care hospital in Japan. Participants were divided into 2 groups: breastfeeding mothers prescribed tacrolimus (*n* = 6); and breastfeeding mothers who did not receive tacrolimus (control group; *n* = 12). Most infants were primarily breastfed. Between January 2011 and June 2018, maternal characteristics and adverse events in infants from birth to the 1-month checkup were collected from medical records and “plan-sheets for lactation” completed at approximately 28 weeks’ gestation.

**Results:**

All mothers in the tacrolimus group received a daily tacrolimus dose of 3 mg throughout the observation period. The adverse events observed in the tacrolimus group included jaundice (*n* = 5), rashes (*n* = 3), vomiting (*n* = 3), head hematoma (*n* = 2), minor skin lesion (*n* = 1), and respiratory failure (*n* = 1). All adverse events were non-serious and the infants recovered uneventfully. The Isobe Neonatal Withdrawal Syndrome score was 0 in all evaluated infants in the tacrolimus group during the first month after birth. All 6 mother-infant pairs in the tacrolimus group continued breastfeeding and maternal medication through the 1-month postpartum checkup. A significantly higher incidence of vomiting was observed in the tacrolimus group compared with the control group.

**Conclusion:**

Preliminary observations in this small Japanese cohort revealed no serious adverse events in breastfed infants during the first month, but larger studies are required to confirm safety.

## Background

Autoimmune diseases, including systemic lupus erythematosus (SLE), are prevalent among women of childbearing age and disease activity can increase during pregnancy [1]. Tacrolimus is an immunosuppressive agent classified as a calcineurin inhibitor, which modulates immune responses by suppressing T-cell activation. It is commonly used in organ transplantation and selected autoimmune conditions. Tacrolimus is occasionally prescribed to pregnant women as an important therapeutic option for management of autoimmune diseases [2].

In patients diagnosed with rheumatoid arthritis, tacrolimus has been reported to have a prolonged blood concentration half-life (34.89 ± 8.69 h) [3]. This indicates that tacrolimus may remain in the mother’s bloodstream after delivery even when its concentration is maintained within the therapeutic range. Additionally, tacrolimus metabolism is influenced by drug-metabolizing enzymes, particularly cytochrome P450 (CYP) 3A5, the activity of which varies due to genetic polymorphisms [4]. Individuals classified as poor metabolizers (CYP3A5*3/*3) exhibited an area under the curve for tacrolimus that was 1.8 times greater compared with intermediate or extensive metabolizers (carriers of the CYP3A5*1 allele). In Japanese, more than half of individuals are classified as CYP3A5 poor metabolizers, and in such cases, the elimination half-life of tacrolimus has been reported to extend up to 60 h [5, 6]. Furthermore, relative bioavailability was 83% higher in Asians, including Japanese, than in Whites (90% confidence interval (CI): 59%–210%) and 51% higher than in Blacks (90% CI: 32%–74%) [7]. Although investigations in Japanese populations are warranted, reports remain limited.

In Japan, tacrolimus package inserts were revised in 2018 to remove pregnancy and breastfeeding from the list of contraindications. However, this revision was based on studies conducted in non-Japanese populations. Differences in drug-metabolizing enzyme expression and breast milk composition across racial groups suggest that further research is required to assess their safety in Japanese women [8, 9].

Our previous study [10] demonstrated the safety of tacrolimus use during pregnancy, but did not evaluate its safety during breastfeeding. For patients with autoimmune diseases, it is essential to make informed and strategic decisions regarding medication use to ensure safety, not only during pregnancy but also during the postpartum breastfeeding period. The present study, therefore, aimed to expand on the available evidence and assess the safety of tacrolimus administration in breastfeeding Japanese mothers.

## Methods

### Plan-sheets for lactation

In January 2011, a systematic evaluation of the risk in infants, whose mothers breastfed while taking drugs at the Department of Obstetrics, Tohoku University Hospital (Miyagi, Japan) [11, 12], was commenced. Initially, pregnant women were asked to provide information including disease name, symptoms, drug use, dosage regimen, and desire for breastfeeding after delivery on the check-sheets, termed “plan-sheets for lactation”, at approximately 28 weeks’ gestation. Based on the check-sheets, midwives interviewed each pregnant woman about her background and noted their comments as mothers’ concerns and requirements regarding breastfeeding after delivery; these check-sheets were sent to the Department of Pharmaceutical Sciences. Pharmacists collect information regarding the safety of the drugs administered to these women during lactation from sources such as *Medications and Mothers’ Milk* [13] and *Drugs in Pregnancy and Lactation* [14], and then provide this information to obstetricians. Obstetricians discussed the risks of breastfeeding for infants by mothers taking drugs during the lactation period with pediatricians and explained the results of the medical staff’s evaluation of the pregnant women.

### Data collection and evaluation

Pregnant women with autoimmune diseases receiving tacrolimus, who were evaluated using a lactation plan-sheet between January 2011 and June 2018, were enrolled. The exclusion criteria were as follows: women who delivered at another hospital; women who discontinued breastfeeding before the 1-month infant check-up; infants who were exclusively formula-fed; and women who discontinued tacrolimus the day before delivery. Maternal characteristics, including age, daily dosage, indication for tacrolimus, other drug prescriptions, and gestational age at delivery, were collected from medical records and the plan sheet for lactation. In addition, infant feeding status was documented from the medical records. Although the precise proportion of breast milk versus formula was not recorded, most infants were primarily breastfed.

Infant characteristics were also retrospectively collected from medical records, including sex, birth weight (BW), feeding method (exclusive breastfeeding or mixed feeding), the Isobe Neonatal Withdrawal Syndrome (NWS) score [15], and adverse events (AEs) that occurred from birth to 1 month after delivery. The NWS is a modified version of the Finnegan score [16], which is a point-based system designed for early detection and treatment of drug withdrawal symptoms. The Isobe scoring system primarily evaluates symptoms related to the digestive, autonomic, and central nervous systems. An NWS score ≥ 8 generally indicates the need for medical intervention [15]. Infant symptoms were routinely evaluated by medical staff at 2 h, 1–6 days, 2 weeks, and 1 month after delivery, and the highest score recorded during this period was used as the NWS score for analysis.

Women diagnosed with autoimmune disease, who were not administered tacrolimus and were evaluated using plan sheets between April 2017 and March 2018, were enrolled as the source population for the control group. Patients in the control group were selected using the same exclusion criteria as the tacrolimus group. After one-to-two matching with the tacrolimus-treated group according to BW and gestational age at birth, they were included in the control group. The 2 groups were not matched for the distribution of underlying maternal diseases or concomitant medications, which may act as potential confounders. Characteristics of the mothers and infants in the groups were compared using the unpaired Mann–Whitney U test and Fisher’s exact test, as appropriate. Differences with *p* < 0.05 were considered to be statistically significant. For proportions of adverse events, 95% CI were calculated using the Clopper-Pearson exact method to account for the small sample size.

## Results

### Enrollment of the tacrolimus group

Nine pregnant women who were evaluated using a lactation plan sheet were enrolled. Two women were excluded due to tacrolimus discontinuation the day before delivery. One additional woman discontinued breastfeeding before the 1-month check-up to begin warfarin therapy. Ultimately, 6 women were included in the tacrolimus group.

### Maternal characteristics of the tacrolimus group

Maternal characteristics are summarized in Table 1. None of the mothers were of advanced maternal age (≥ 35 years). All participants received the same daily dose of tacrolimus (3 mg, once daily). All 6 women were also prescribed prednisolone, with 4 receiving daily doses ≥ 10 mg; no other corticosteroids were administered. SLE was the most common indication for tacrolimus therapy. None of the mothers discontinued tacrolimus treatment within 1 month postpartum due to AEs in their infants.

**Table 1:** Maternal and infants’ characteristics of tacrolimus group

No.	Characteristics of Mothers				Characteristics of Infants									
	Age (years)	Purpose	Gestational weeks at delivery		Sex	Body weight (g)			Nutrition methods	NWS score	Adverse events			Comments
						At birth	At one-month	Weight gain			Vomiting	Skin	JD	
1	30s	ITP	38		M	2,482	No data	No data	Mix	No data			+	
2	20s	SLE	36		F	2,654	3,262	608	EBF	0		+	+	She was born with a head hematoma.
3	30s	SLE	38		M	2,686	3,830	1,144	Mix	No data	+	+	+	He was born with a head hematoma
4	20s	SLE	38		M	2,828	4,034	1,206	Mix	0				
5	30s	SLE	35		M	2,198	3,636	1,438	Mix	0	+	+	+	Due to hypoxia at delivery, oxygen administration is used for infant at day 0–2.
6	20s	SLE	37		F	2,176	3,378	1,202	Mix	No data	+	+	+	At day 5, there was minor skin lesion in her axilla, and zinc oxide (10%) simple ointment were prescribed.

All mothers were administrated 3 mg of tacrolimus. EBF: Exclusive breastfeeding, F: Female, ITP: Idiopathic thrombocytopenic purpura, JD: Jaundice, M: Male, Mix; Mixed feeding, NWS: Neonatal withdrawal syndrome, Skin: Skin symptom, SLE: Systemic lupus erythematosus

### Characteristics of infants in the tacrolimus group

Infant characteristics are summarized in Table 1. Six infants were delivered during the study period, all of which were singleton pregnancies. Two infants were born preterm, and 3 had low BW. Body weight data at the 1-month checkup were unavailable for 1 infant (No. 1). Among the others, 1 infant (No. 2) gained 22.9% of her BW, while the remaining 4 (Nos. 3–6) exhibited an average weight gain of 51.5% (range, 42.6%–65.4%) from birth to the 1-month follow-up.

NWS scores were recorded for 3 of the 6 infants, with all scores < 8 points, whereas scores were unavailable for the remaining infants. AEs observed within the first month included jaundice (*n* = 5), skin symptoms, such as rashes (*n* = 3), minor skin lesion (*n* = 1), and vomiting (*n* = 3). Vomiting was attributed to the overconsumption of breast milk according to medical records. One infant (No. 5) required oxygen therapy for respiratory failure, and another (No. 6) was prescribed a zinc oxide ointment for minor skin lesion. Although the exact size of the skin lesion was not documented, it did not require specialist consultation and resolved with conservative management.

### Comparison of the tacrolimus and control groups

Twelve mother-infant pairs were included in the control group (Table 2). Except for gestational weeks at delivery, no significant differences were found between the groups in terms of maternal or infant baseline characteristics (Table 3). None of the mothers in either group were administered medications categorized as L4 (possibly hazardous) or L5 (contraindicated) according to *Medications and Mothers’ Milk* [13]. Similarly, none of the mothers were prescribed teratogenic or fetotoxic drugs including angiotensin-converting enzyme inhibitors, angiotensin II receptor blockers, aminoglycoside antibiotics, carbamazepine, cyclophosphamide, danazol, etretinate, methotrexate, misoprostol, mycophenolate, phenobarbital, phenytoin, tetracycline antibiotics, thalidomide, thiamazole, trimethadione, valproate, vitamin A (retinol), or warfarin [17]. With regard to infant outcomes, a significantly higher incidence of vomiting was observed in the tacrolimus group than that in the control group (Table 3).

**Table 2:** Maternal and infants’ characteristics of control group

No.	Characteristics of Mothers				Characteristics of Infants									
	Age (years)	Purpose	Gestational weeks at delivery		Sex	Body weight (g)			Nutrition methods	NWS score	Adverse events			Comments
						At birth	At one-month	Weight gain			Vomiting	Skin	JD	
1	30s	AOSD	36		M	1,710	2,694	984	Mix	No data		+		
2	30s	GD	39		M	2,950	3,660	710	Mix	No data				After giving birth, the mother stopped taking potassium iodide.
3	40s	GD	41		F	3,484	4,478	994	Mix	No data		+	+	
4	20s	GD	39		M	3,026	4,364	1,338	Mix	0		+	+	
5	30s	CD	38		F	3,110	4,125	1,015	Mix	No data				
6	30s	GD	39		M	3,412	4,734	1,322	Mix	No data				
7	30s	MS	40		F	3,198	3,962	764	Mix	No data		+	+	
8	20s	SLE, APS	38		F	2,412	3,250	838	Mix	No data		+	+	
9	20s	GD	38		F	2,386	3,576	1,190	Mix	No data			+	
10	30s	GD	40		M	3,062	4,228	1,166	Mix	No data				
11	20s	SLE	38		M	2,804	4,516	1,712	Mix	No data				
12	30s	ITP	37		F	2,680	4,374	1,694	Mix	No data		+	+	

AOSD: Adult-onset Still’s disease, APS: Anti-phospholipid antibody syndrome, CD: Crohn’s disease, F: Female, GD: Graves’ disease, ITP: Idiopathic thrombocytopenic purpura, JD: Jaundice, M: Male, Mix; Mixed feeding, MS: Multiple sclerosis, NWS: Neonatal withdrawal syndrome, Skin: Skin symptom, SLE: Systemic lupus erythematosus

**Table 3:** Comparison of tacrolimus group and control group

	Tacrolimus group (*n* = 6)	Control group (*n* = 12)	*p*-value
Mothers			
Age (≥ 30 years old) (n [%], 95%CI)	3 (50.0, 11.8–88.2)	8 (66.7, 34.9–90.1)	0.627
Diagnosis (n [%])			
Adult-onset Still’s disease	0 (0)	1 (8.3)	
Anti-phospholipid antibody syndrome	0 (0)	1 (8.3)	
Crohn’s disease	0 (0)	1 (8.3)	
Graves’ disease	0 (0)	6 (50.0)	
Idiopathic thrombocytopenic purpura	1 (16.7)	1 (8.3)	
Multiple sclerosis	0 (0)	1 (8.3)	
Systemic lupus erythematosus	5 (83.3)	2 (16.7)	
Infants			
Sex (male) (n [%], 95%CI)	4 (66.7, 22.3–95.7)	3 (25.0, 5.5–57.2)	0.141
Body weight (gram, min-max)			
At birth (A)	2,508 (2,176-2,828) *	2,843 (1,710-3,484)	0.103
At 1-month checkup (B)	3,628 (3,378-4,034) *	3,978 (2,694-4,734)	0.156
Weight gain (B − A)	1,120 (608-1,438) *	1,135 (710-1,712)	0.873
Low birth weight (< 2,500 g) (n [%], 95%CI)	3 (50.0, 11.8–88.2)	3 (25.0, 5.5–57.2)	0.344
Gestational weeks at delivery (weeks, min-max)	37.0 (35–38)	38.6 (36–41)	0.044
Preterm birth (n [%], 95%CI)	2 (33.3, 4.3–77.7)	1 (8.3, 0.2–38.5)	0.245
Nutrition methods (Exclusive breastfeeding) (n [%], 95%CI)	1 (16.7, 0.4–64.1)	0 (0, 0-26.5)	0.333
Presence of adverse events (n [%], 95%CI)			
Vomiting	3 (50.0, 11.8–88.2)	0 (0, 0-26.5)	0.025
Skin symptom	4 (67.7, 22.3–95.7)	6 (50.0, 21.1–78.9)	0.638
Jaundice	5 (83.3, 35.9–99.6)	6 (50.0, 21.1–78.9)	0.316

* Analysis was performed excluding infant No. 1 in Table 1 (No data was abailable on body weight at 1-month checkup)

CI: confidencial interval

## Discussion

In this study, we evaluated the safety of tacrolimus during lactation. There have been case reports addressing the safety of infants breastfed by Japanese mothers who received tacrolimus for the treatment of autoimmune diseases [18, 19]. In both cases, the infants were healthy. However, the sample sizes in these reports were small, and further investigation is required to confirm safety in infants.

A nationwide study reported that approximately 10% of singleton pregnancies in Japan resulted in low-BW infants [20]. In our study, the incidence of low BW in the control group was 25% and even higher in the tacrolimus group. The tacrolimus group had a significantly shorter gestational age at delivery, although preterm birth rate did not differ significantly between the groups. These observations may reflect the influence of underlying maternal conditions, particularly SLE, and concomitant medications such as high-dose prednisolone, which are known risk factors for preterm birth and low BW [21, 22]. In addition, this trend may, in part, be explained by previous studies reporting that infants born to mothers receiving immunosuppressive therapy after organ transplantation were at increased risk for low BW and preterm birth [23, 24]. Given the small sample size, the study may have been underpowered to detect differences in preterm birth rates or to disentangle the relative contributions of maternal disease severity and tacrolimus exposure. Therefore, a potential effect of tacrolimus on gestational age or BW cannot be entirely excluded. These findings should be interpreted cautiously, considering the possible confounding effects of maternal health status and concomitant therapy. Further studies with larger cohorts are needed to clarify these associations.

We collected data regarding changes in infant weight during the first month after birth. According to a prospective birth cohort study [25], the average weight gain at 1 month postpartum was approximately 1098.7 g, which is comparable with our results. However, 1 infant (No. 2) in the tacrolimus group exhibited a weight gain of only 608 g during the study period (Table 1). Although the infant’s weight at the 1-month checkup was within the normal range for Japanese female infants (2905–4838 g, third to 97th percentile) [26], no specific reason for the limited weight gain was noted in the medical records. The infant was exclusively breastfed and bottle feeding was not practiced. Additionally, the mother was prescribed a high dose of prednisolone (30 mg/day orally), which reduced milk volume [27]. Tacrolimus also been shown to lower prolactin levels, potentially affecting milk production [28]. These factors may have contributed to the lower weight gain observed among the infants. Mixed feeding may be considered in cases of insufficient weight gain.

In the present study, 5 infants in the tacrolimus group experienced AEs (Table 1). One infant (No. 5) developed respiratory failure requiring oxygen administration, which was attributed to perinatal hypoxia rather than tacrolimus exposure through breast milk. The infant was transferred from the neonatal intensive care unit to the mother’s room on day 2 postpartum. Four infants experienced skin symptoms, including rashes (*n* = 3) and minor skin lesion (*n* = 1). The minor skin lesion treated with zinc oxide ointment was considered to be mild, and no referrals to dermatologists were necessary. No significant differences in the incidence of AEs were observed between the tacrolimus and control groups, suggesting that these symptoms were unrelated to tacrolimus administration.

Vomiting was observed in three infants in the tacrolimus group, with a significantly higher incidence than in the control group. According to the medical records, this symptom was attributed to overconsumption of breast milk rather than to tacrolimus exposure. Nevertheless, the possibility that tacrolimus contributed to gastrointestinal symptoms cannot be completely ruled out given the small sample size. The infants who experienced vomiting showed no problems with postnatal weight gain in this study; however, careful monitoring of gastrointestinal symptoms and infant weight gain is recommended, and formula supplementation may be considered if breastfeeding is insufficient.

The incidence of jaundice in Japanese newborns has been reported to be 57.39% [29]. The incidence of jaundice in the control group was consistent with previously reported values. In contrast, 5 (83.3%) infants in the tacrolimus group developed jaundice, with 1 requiring phototherapy. Although the difference between the groups was not statistically significant, the higher prevalence observed in the tacrolimus group suggests a possible contribution of tacrolimus exposure during lactation, which cannot be completely excluded.

Although these drugs are known to induce renal dysfunction, there were no indications in the medical records of symptoms suggestive of renal dysfunction, such as edema or decreased urine output, in any infant. Additionally, two infants had head hematomas that were neither treated nor followed. We believe that these symptoms are unlikely to be related to tacrolimus exposure in the breast milk.

There were differences in the maternal diagnoses between the 2 groups. Most mothers in the tacrolimus group had SLE, which is associated with autoantibodies such as anti-Ro/SSA and anti-La/SSB [30]. Neonatal lupus erythematosus (NLE), a condition associated with these antibodies, presents as a skin rash. Although none of the infants in our study were diagnosed with NLE, the increased skin symptoms observed in the tacrolimus group could be related either to maternal SLE or to tacrolimus exposure.

In this study, only 3 of the 6 infants had an NWS score of 0. Tacrolimus is not listed as a drug that requires attention for NWS in “the manual for handling disorders due to adverse drug reactions” [31]. Furthermore, no reports of withdrawal symptoms associated with tacrolimus have been documented and such symptoms were not observed in this study. These findings suggest that tacrolimus use during lactation is not associated with NWS, thereby supporting its relative safety.

### Limitations

The present study had several limitations. First, we could not systematically collect data regarding tacrolimus blood concentration in infants. Because blood sampling is invasive, routine measurements are impractical. Additionally, blood concentration measurements were clinically unnecessary because no severe AEs were observed. Previous report in a single Japanese mother-infant pair measured tacrolimus concentration in their blood and breast milk and showing a low breast milk/maternal blood ratio of 0.4–0.64 [32]. Based on the findings, tacrolimus concentrations were unlikely to be high in the breast milk in our cases either. Nonetheless, individual variability cannot be excluded. Moreover, as urine tests and serum creatinine test were not performed, we were unable to assess the potential occurrence of acute renal injury, which is a known adverse effect of tacrolimus. Second, this study relied on data recorded up to the 1-month check-up, and long-term safety could not be assessed. After this 1-month check-up visit, infants were referred back to their original medical institutions, and none were subsequently re-referred to Tohoku university hospital; however, long-term safety cannot be confirmed from these data. Third, although all eligible cases during the study period were included, the sample size was very small because only a limited number of mothers who were prescribed tacrolimus breastfed their infants. As a result, rare AEs may not have been detected. This study should be considered exploratory, providing preliminary insights into the safety of tacrolimus during breastfeeding in Japanese women. Our data must be interpreted carefully, and further research with larger cohorts is required. Finally, although most infants were primarily breastfed, the exact proportion of breast milk versus formula intake was not quantified. Therefore, the actual extent of tacrolimus exposure via breast milk could not be precisely assessed, which limits interpretation of observed AEs such as vomiting or changes in weight gain.

## Conclusion

In this exploratory study, no serious AEs were detected in breastfed infants during the first month postpartum, although the small sample size precludes firm conclusions regarding rare AEs. While our findings are consistent with previous reports, further research is needed to clarify long-term safety in larger Japanese cohorts.

## Data Availability

The datasets generated during the current study are not publicly available due to participant confidentiality.

## Abbreviations

AE: Adverse event
BW: Birth weight
CI: Confidence interval
CYP: Cytochrome P450
NLE: Neonatal lupus erythematosus
SLE: Systemic lupus erythematosus
NWS: Neonatal Withdrawal Syndrome
